# Leptin and Melanocortin Signaling Mediates Hypertension in Offspring From Female Rabbits Fed a High-Fat Diet During Gestation and Lactation

**DOI:** 10.3389/fphys.2021.693157

**Published:** 2021-06-24

**Authors:** Kyungjoon Lim, Sandra L. Burke, Francine Z. Marques, Kristy L. Jackson, Cindy Gueguen, Yusuke Sata, James A. Armitage, Geoffrey A. Head

**Affiliations:** ^1^Neuropharmacology Laboratory, Baker Heart and Diabetes Institute, Melbourne, VIC, Australia; ^2^Department of Physiology, Anatomy and Microbiology, La Trobe University, Bundoora, VIC, Australia; ^3^Heart Failure Research Group, Baker Heart and Diabetes Institute, Melbourne, VIC, Australia; ^4^Hypertension Research Laboratory, School of Biological Sciences, Faculty of Science, Monash University, Clayton, VIC, Australia; ^5^Human Neurotransmitters Laboratory, Baker Heart and Diabetes Institute, Melbourne, VIC, Australia; ^6^Faculty of Medicine, Nursing and Health Sciences, Central Clinical School, Monash University, Melbourne, VIC, Australia; ^7^Department of Cardiology, Alfred Hospital, Melbourne, VIC, Australia; ^8^School of Medicine (Optometry), and IMPACT Institute for Innovation in Physical and Mental Health and Clinical Translation, Faculty of Health, Deakin University, Waurn Ponds, VIC, Australia; ^9^Department of Pharmacology, Monash University, Clayton, VIC, Australia

**Keywords:** programming, neuronal plasticity, hypertension, maternal high-fat diet, sympathetic nerve activity

## Abstract

Maternal high-fat diet in rabbits leads to hypertension and elevated renal sympathetic nerve activity (RSNA) in adult offspring but whether this is due to adiposity or maternal programming is unclear. We gave intracerebroventricular (ICV) and ventromedial hypothalamus (VMH) administration of leptin-receptor antagonist, α-melanocyte-stimulating hormone (αMSH), melanocortin-receptor antagonist (SHU9119), or insulin-receptor (InsR) antagonist to conscious adult offspring from mothers fed a high-fat diet (mHFD), control diet (mCD), or mCD offspring fed HFD for 10d (mCD10d, to deposit equivalent fat but not during development). mHFD and mCD10d rabbits had higher mean arterial pressure (MAP, +6.4 mmHg, +12.1 mmHg, *p* < 0.001) and RSNA (+2.3 nu, +3.2 nu, *p* < 0.01) than mCD, but all had similar plasma leptin. VMH leptin-receptor antagonist reduced MAP (−8.0 ± 3.0 mmHg, *p* < 0.001) in mCD10d but not in mHFD or mCD group. Intracerebroventricular leptin-receptor antagonist reduced MAP only in mHFD rabbits (*p* < 0.05). Intracerebroventricular SHU9119 reduced MAP and RSNA in mHFD but only reduced MAP in the mCD10d group. VMH αMSH increased RSNA (+85%, *p* < 0.001) in mHFD rabbits but ICV αMSH increased RSNA in both mHFD and mCD10d rabbits (+45%, +51%, respectively, *p* < 0.001). The InsR antagonist had no effect by either route on MAP or RSNA. Hypothalamic leptin receptor and brain-derived neurotrophic factor (*BDNF*) mRNA were greater in mHFD compared with mCD rabbits and mCD10d rabbits. In conclusion, the higher MAP in mHFD and mCD10d offspring was likely due to greater central leptin signaling at distinct sites within the hypothalamus while enhanced melanocortin contribution was common to both groups suggesting that residual body fat was mainly responsible. However, the effects of SHU9119 and αMSH on RSNA pathways only in mHFD suggest a maternal HFD may program sympatho-excitatory capacity in these offspring and that this may involve increased leptin receptor and *BDNF* expression.

## Introduction

The obesity epidemic has been on the rise in the past several decades, contributing to a socioeconomic and health burden around the world (Bluher, 2019). Alarmingly, the prevalence of childhood and youth obesity has grown more than in older age groups (NCD-RisC, 2017). While there is clear evidence of a correlation between maternal and offspring obesity with the development of hypertension (Lurbe et al., 2018; Taylor et al., 2018; Kislal et al., 2020), the mechanisms underlying this relationship are still unclear. Importantly, approximately 30% of all pregnant women are considered to be obese thereby exposing their children to an obesogenic intrauterine environment throughout the course of gestation (Desai et al., 2013). This exposure to over-nutrition during development greatly increases the prevalence of offspring obesity and obesity-related diseases, such as metabolic syndrome, insulin resistance, leptin resistance, and hypertension (Zambrano et al., 2016; Kislal et al., 2020). Reports from both clinical (Glastras et al., 2018) and experimental studies (Taylor et al., 2014; Bringhenti et al., 2015) have clearly suggested that maternal obesity *per se* has adverse effects on long-term cardiovascular function in the child and is associated with an increase in all-cause mortality (Lee et al., 2015) and cardiometabolic morbidity in adulthood (Kislal et al., 2020).

We have previously shown that feeding pregnant rabbits with a high-fat diet (HFD) led to hypertension, increased adipose deposition, heart rate (HR), and renal sympathetic nerve activity (RSNA) in the offspring in adulthood (Prior et al., 2014). We suggested that these changes were associated with increased sensitivity to leptin in the hypothalamus as well as a contribution from insulin. Plasma leptin crosses the blood–brain barrier to activate receptors in the hypothalamus and brainstem (Dhillon et al., 2001) that stimulate the melanocortin system to not only influence appetite but also blood pressure (BP) *via* SNA (Burguera and Couce, 2001; Shi et al., 2015). The ventromedial hypothalamus (VMH) is a key area of integration (Klockener et al., 2011; Lim et al., 2016) and exposure to an obesogenic environment during early development may program the VMH of adult offspring to exhibit changes in the sensitivity to leptin and insulin, leading to hypertension and sympatho-excitation (Prior et al., 2014). We have also shown that a short period of HFD in adult rabbits lead to increased BP and RSNA as well as increased activation of melanocortin pathways much like in the programmed rabbits (Prior et al., 2010; Lim et al., 2013, 2016; Barzel et al., 2016).

In the current study, we addressed whether it is programmed or adult-acquired adiposity that is the origin of the hypertension and sympatho-excitation observed in adult offspring of mothers fed a HFD (mHFD) during pregnancy and lactation. We therefore compared the effects of a mHFD in conscious adult offspring with short-term feeding of HFD in adulthood (10 days on HFD) on BP and RSNA. The fat accumulated after 10 days of HFD during adulthood was thus in excess (“residual”) of the fat accumulated in utero and during lactation. We also determined whether the sensitivity of pathways associated with pro-opiomelanocortin neurons located in the central nervous system, specifically the VMH, was altered in offspring of mHFD rabbits using both intracerebroventricular (ICV) injections and direct administration in the VMH of either a leptin-receptor (LepR) antagonist, α-melanocortin-stimulating hormone (αMSH), melanocortin 3 and 4 receptor (MC3/4R) antagonist SHU9119, or insulin-receptor (InsR) antagonist. To further identify the pathways involved, we examined mRNA expression of relevant signaling molecules in the hypothalamus including brain-derived neurotrophic factor (*BDNF*) which is associated with obesity-induced hypertension and sympathetic activation (Barzel et al., 2016).

## Materials and Methods

### Animals

**Figure 1:**
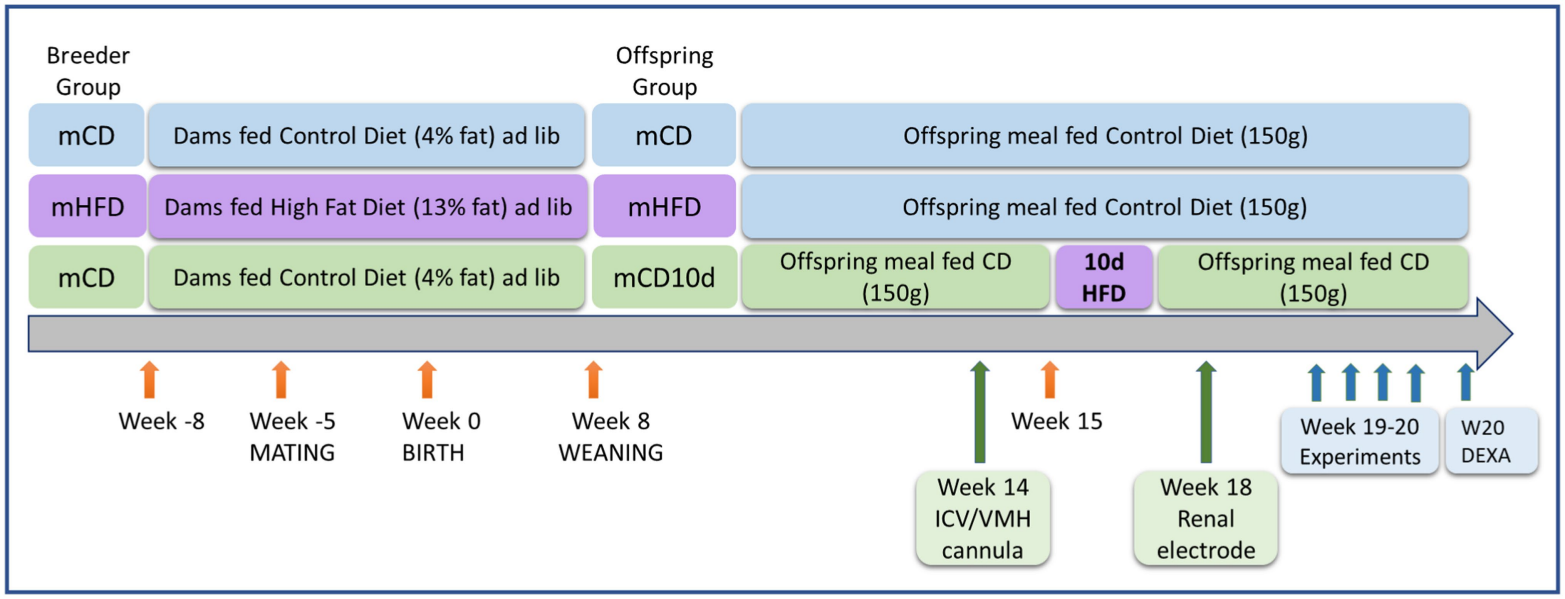
Schematic diagram showing timeline of dietary treatments, surgery, and experiments. Ages and life events are shown with orange arrows for dams fed a control diet (CD) or high-fat diet (HFD) and their offspring. Surgery to implant the ICV or VMH cannula and renal electrode is shown with green arrows. Experiments to determine the effects of agonists and antagonists and to measure adipose tissue are shown with blue arrows.

Five male and 11 female New Zealand White rabbit breeders (initial body weight 2.6–3.1 kg) were housed individually under controlled light (lights on 6:00 AM to 6:00 PM) and temperature (22 ± 2°C) with food and water *ad libitum*. Male breeders were fed a control diet (CD) containing 2.63 kcal/g, of which 4.0% of calories were from fat, 17.8% protein, 15% crude fiber, and 19% acid detergent fiber (Specialty Feeds, Glen Forrest, WA, Australia). Females were fed a HFD (3.34 kcal/g, of which 13.4% of calories were from fat, 17.5% protein, 13.1% crude fiber, and 16% from acid detergent fiber, Specialty Feeds) or a CD, for 3 weeks prior to mating, during pregnancy and for a further 8 weeks after birth until weaning (Figure 1; Prior et al., 2010, 2014). Offspring from mHFD were meal-fed once per day a CD (150 g) after weaning. Offspring from maternal CD-fed rabbits (mCD) at 15 weeks of age were then fed either a HFD (meal-fed 190 g) for 10 days and then a CD for 3 weeks until the end of the experimentation period (mCD10d) or a CD throughout (Figure 1). In total, 103 18–20 week-old offspring (32 mCD, 16 female, 16 male; 39 mHFD, 24 female, 15 male; and 32 mCD10d, 21 female, 11 male) were used. Experiments were approved by the Alfred Medical Research Education Precinct Animal Ethics Committee in accordance with the Australian Code for Care and Use of Animals for Scientific Purposes.

### Surgical Procedures

Rabbits underwent two operations under isoflurane anesthesia (3–4% in 1 L/min oxygen) following induction with propofol (10 mg/kg iv, Fresenius Kabi, Pymble, NSW, Australia). The analgesic carprofen (3 mg/kg sc, Pfizer, North Ryde, NSW, Australia) was given 30 min beforehand and 24 h after surgery. The operations were conducted at 14 and 18 weeks of age (Figure 1). Initially an ICV cannula (Plastics One, Roanoke, VA, United States) was implanted into the lateral ventricle (3 mm lateral and 4 mm ventral to bregma; Head and Williams, 1992) or a bilateral brain cannula (22-gauge, Plastics One) was positioned 2 mm above the VMH (2.2 mm caudal to bregma, ±0.9 mm lateral to midline at 16 mm below the skull; Lim et al., 2016). Secondly, a renal nerve electrode was implanted at week 18 (Dorward et al., 1985), 1 week prior to the first experiment (Figure 1). Briefly, the electrode was implanted *via* a retroperitoneal incision under isoflurane anesthetic. The renal nerves were gently separated from underlying tissue, inserted into the two stainless steel wire coils of the electrode and embedded in a silicone elastomer (Kwik-sil, World Precision Instruments, Sarasota, Florida, United States). The plug at the other end of the electrode was buried under the skin for later retrieval. The rabbit was allowed 7-days recovery before the first experiment. As each rabbit underwent four conscious experiments with at least 1-day recovery in between, the electrode was in place for approximately 14 days.

### Plasma Collection and Biochemical Analysis

Animals were fasted for 12 h before blood samples were collected. In mCD and mHFD rabbits, blood was collected at euthanasia (19–20 weeks of age) which corresponded to 3 weeks after the 10d period of HFD feeding in mCD10d rabbits. Arterial blood (3 ml) was drawn into vacuum sealed cylinders containing K3EDTA (Vacuette Premium, Greiner Bio-one, Wemmel, Belgium) and spun at 4°C for 10 min at 3,000 rpm. Plasma aliquots (200 μl) were snap frozen in liquid nitrogen and stored at −80°C until use. Plasma glucose, triglyceride, high-density lipoprotein (HDL) cholesterol, and low-density lipoprotein (LDL) cholesterol were measured on a Roche Cobas Integra 400 plus autoanalyzer by enzymatic colorimetric methods that used commercially available kits (CHOL2, TRIGL, and Glucose HK Gen 3, Roche Diagnostic, Australia) according to the manufacturer’s instructions. Plasma leptin concentration was assessed using an ultra-sensitive radioimmunoassay multispecies kit (Millipore, Billerica, MA, United States).

### Measurement of Cardiovascular Variables and RSNA

**Figure 2:**
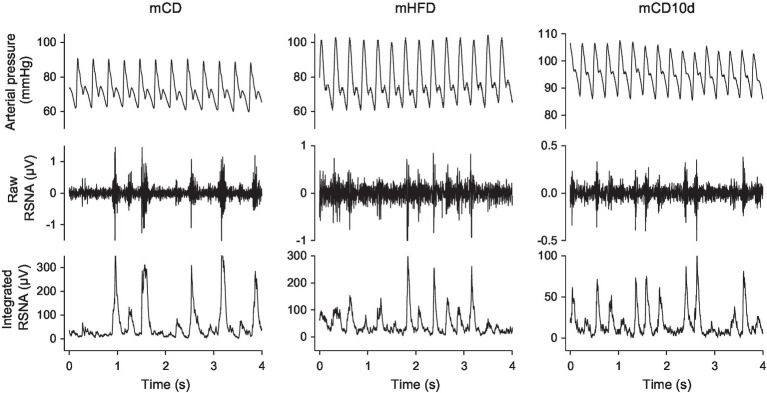
Sections of records from individual mCD, mHFD, and mCD10d rabbits at rest. For each group, arterial pressure (top), raw renal sympathetic nerve activity (RSNA, middle), and integrated RSNA (bottom) are shown.

Experiments were conducted between weeks 19 and 20 in conscious rabbits held in a rabbit holding box (Figure 1). Mean arterial pressure (MAP) was measured from the central ear artery *via* a 22G transcutaneous catheter (BD Insyte, Singapore) connected to a pressure transducer (Statham P23DG transducer; Hato Rey, Puerto Rico; Burke and Head, 2003). The plug of the renal electrode was retrieved under local anesthetic and connected to the recording cable and after the experiment, the plug was returned under the skin. MAP and RSNA were digitized at 500 Hz using a data acquisition card (National Instruments 6024E, Austin, TX, United States). The RSNA signal was rectified and integrated using a 20-min time constant and burst amplitude and frequency were also measured (Figure 2; Burke et al., 2016). Rabbits were allowed 30-min recovery from handling before commencing the experiment to allow recorded parameters to stabilize.

### ICV and VMH Dose Response and Antagonist Experiments

Either vehicle (Ringer’s solution, Baxter, Old Toongabbie, NSW, Australia) or one of the four drugs (maximum four in one rabbit) were administered in random order on separate days with 1-day recovery in between.

For ICV experiments, after 1 h baseline recording, an initial 50 μl ICV injection of vehicle was given using an injector 2 mm longer than the guide cannula (28-gauge, Plastics One). Increasing doses of either αMSH (1, 3, 10 nmol in 50 μl, Tocris, Ellisville, MI, United States) or SHU9119 (0.0375, 0.075, 0.185, and 0.375 nmol/50 μl, Tocris) were injected 30 min apart. A single dose of LepR antagonist (mouse leptin antagonist LAN-3 gene ID 16846, 100 μg/50 μl, Protein Laboratories Rehoboth, Rehovot, Israel) or InsR antagonist (NNC0069-0961, 0.5 IU in 50 μl, S961, Novo Nordisk, Baulkham Hills, NSW, Australia) was also given, the doses determined in our previous study (Barzel et al., 2016).

For VMH experiments, a 1 h baseline recording was followed by bilateral administration of two doses of each drug in a volume of 400 nl in the VMH over 30s. Either the LepR antagonist (5, 10 μg, Protein Laboratories), αMSH (0.3, 1 nmol, Tocris), SHU9119 (0.0185, 0.0375 nmol, Tocris), or InsR antagonist (0.01, 0.05 IU, Novo Nordisk) was injected, and doses were given 60 min apart using an injector 4 mm longer than the guide cannula (28-gauge, Plastics One; Lim et al., 2016). A time control study on a separate day involved 400 nl injections of Ringer’s solution. The order of all drug treatments, including Ringer’s solution, was randomized and 1-day recovery allowed in between.

### Tissue Collection, Assessment of Body Composition, and Confirmation of Cannula Placement

**Figure 3:**
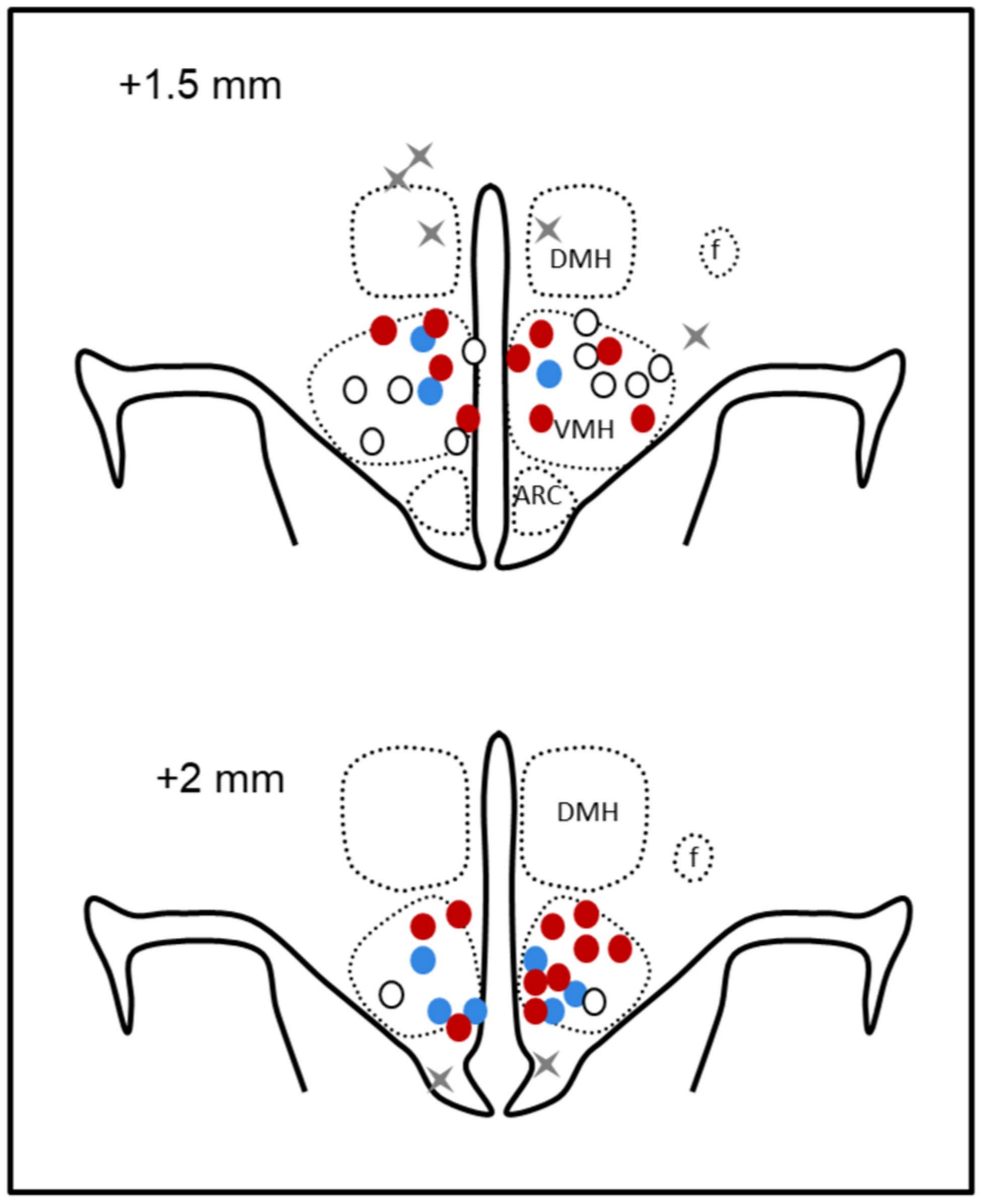
Schematic representation of microinjection sites. Coronal sections at 1.5 mm and 2 mm posterior to bregma showing bilateral microinjection sites as circles in offspring of mCD (white), maternal HFD (red), and mCD with 10 days HFD (blue) when in the ventromedial hypothalamus and gray crosses when outside (*n* = 4). DMH, dorsomedial hypothalamus; f, fornix; and arc, arcuate nucleus.

Rabbits were killed by 160 mg/kg sodium pentobarbitone (i.v., Virbac, Milperra, NSW, Australia). White adipose tissue (WAT) was dissected from the perirenal, omental and epididymal areas, and between the scapulae and weighed. Lean muscle mass, bone mineral content, and total % of fat were determined by scanning with a bone densitometer (DEXA, Dual-Energy X-Ray Absorptiometry, Hologic Discovery A-QDR Inc., MA, United States). VMH cannula placements were verified by injection of methylene blue dye before perfusion fixation of the brain and sectioning in a cryostat. Four rabbits with injection sites outside of the VMH were excluded from the study (Figure 3).

### Measurement of Key mRNA Levels in the Hypothalamus (Real-Time PCR)

Fresh hypothalamic tissue was taken from a cohort of rabbits whose brains were not perfusion fixed and stored at −80°C. TRIzol reagent (Life Technologies, Mulgrave, Vic, Australia) was used for RNA extraction from the hypothalamus following the manufacturer’s recommendations. Complementary DNA synthesis was performed using the High-Capacity cDNA Reverse Transcription Kit for total cDNA (Life Technologies). Amplification reactions used the TaqMan^®^ Fast Advanced Master Mix (Life Technologies). Samples were run in duplicates. A quantitative real-time PCR system (model ViiA^™^ 7 qPCR, Life Technologies) and the ΔΔCT method were used to determine the levels of *LEPR, BDNF*, suppressor of cytokine signaling 3 (*SOCS3*), *MC3/4R*, extracellular signal-regulated kinase-2 (*ERK2*), *INSR*, mitogen-activated protein kinase-kinase1 (*MAP2K1*), and phosphatidylinositol-3-kinase (*PI3K*; Table 1).

**Table 1:** RT-PCR primer sequences.

Primers	Forward primer	Reverse primer	Target gene
*LEPR*	ACAATGGGGCCTGAAATGTTAGA	GGCACATGACACTCACAACG	Oryctolagus cuniculus leptin receptor (*LEPR*)
*BDNF*	AGAAGAAGGGCTGTGGTGTG	CCTGGTGGAACTTTATGAGACC	Oryctolagus cuniculus brain-derived neurotrophic factor (*BDNF*)
*SOCS3*	AGTTGTTGCCTTTCACCCCT	TCCGTGAGACAGGGGACATT	Oryctolagus cuniculus suppressor of cytokine signaling (*SOCS3*)
*MC3R*	GCGTGGTCAACAGCAACTAC	GTGACGTACCTATCGACGGC	Oryctolagus cuniculus melanocortin 3 receptor (*MC3R*)
*MC4R*	CCTTGCTTGCCTCCATTTGC	CCGACCCGTTTCACTGTCAT	Oryctolagus cuniculus melanocortin 4 receptor (*MC4R*)
ERK2 *(Mapk1)*	TTCCAAGGGCTACACCAAGTC	CGGGAAGATGGGTCTGTTGG	Oryctolagus cuniculus mitogen-activated protein kinase 1 (*MAPK1*)
*INSR*	GGAAACTACTCCTTCTACGCCT	AGCTTCCCCTGAGAGATGGT	Oryctolagus cuniculus insulin receptor (*INSR*)
*MAP2K1*	TGTGAAGCCCTCCAACATCC	TCTCGGGCGACATATAAGACC	Oryctolagus cuniculus mitogen-activated protein kinase-kinase 1 (*MAP2K1*)
*PI3K*	TCCTCTGCAAAAAGGCCACT	TCTTGCCGTAAATCATCCCCA	Oryctolagus cuniculus phosphatidylinositol-4,5-bisphosphate 3-kinase catalytic subunit alpha (*PI3KCA*)

### Data Analysis

HR was derived from the pressure pulse, and RSNA was normalized to the maximum RSNA elicited by the nasopharyngeal reflex (100 normalized units, nu; Burke et al., 2016). MAP, HR, and RSNA responses to each ICV dose of αMSH or a single dose of LepR antagonist, InsR antagonist, and vehicle were measured over 90 min (six 15-min periods). The responses to each dose of SHU9119 were measured over 120 min (four 30-min periods). Responses to VMH injections (two doses of each drug or vehicle) were measured over 120 min (eight 15-min periods).

Baseline data were averaged over 1 h at the beginning of each experiment (two 30-min periods C1 and C2) and expressed as mean ± SEM. For ICV experiments, the average control period also included the response to the single injection of Ringer’s solution, averaged over 30 min. The effects of drug treatments were expressed as mean ± SEM or mean difference ± SED from the average control period of each treatment. These were averaged over the entire response for LepR and InsR antagonists, over the highest dose for αMSH and the two highest doses for SHU9119. For VMH experiments, effects of drug treatments were calculated from the average control period of each treatment and averaged over the higher dose. The average response to each drug was also compared with the average response to vehicle over the same time period.

Data were analyzed by a mixed model split plot repeated-measures analysis of variance, which allowed for within-animal and between-animal (group) contrasts. The treatment sums of squares were partitioned into the effect of drug over all doses compared to control within groups (*P*_drug_), the effect of diet between each of the groups (*P*_diet_), and the effect of drug treatment compared to vehicle treatment (*P*_veh_). One-way analysis of variance was used for data collected at a single time point. Type 1 error was controlled using Bonferroni adjustment, and the Greenhouse–Geisser correction was used to adjust for asphericity. A probability of *p* < 0.05 was considered significant.

## Results

### Effect of HFD on Body Composition and Plasma Analysis

Body weights compared at the end of the experimental period (18–20 weeks of age) were not different between mCD offspring and those exposed to HFD during pregnancy and lactation (mHFD; Table 2). However, the body weight of offspring exposed to HFD for 10 days in adulthood was 0.21 kg (7%) greater than that of the other two groups (*P*_diet_ < 0.004; Table 2). Total raw WAT and total fat from DEXA scanning were 20 g (+29%) and 86 g (+24%) greater, respectively, in mHFD rabbits when compared to mCD rabbits (*P*_diet_ < 0.05; Table 2). mCD10d rabbits also had 32.5 g more total WAT (+40%) and 129 g (+33%) more DEXA determined fat compared to mCD rabbits (*P*_diet_ < 0.001; Table 2). These differences between groups were maintained if fat mass was expressed as a percentage of body weight or total fat (Table 2). There were no differences in plasma leptin, glucose, triglyceride, and HDL and LDL cholesterol levels among diet groups (*P*_diet_ > 0.05; Table 2).

**Table 2:** Hemodynamics and renal sympathetic nerve activity (RSNA), body composition, and biochemical measurements.

Variable	mCD	mHFD	mCD10d	mCD vs. mHFD	mCD vs. mCD10d	mHFD vs. mCD10d
*Hemodynamics and RSNA*	*n* = 17	*n* = 27	*n* = 16			
MAP (mmHg)	69.4 ± 0.7	75.8 ± 1.2	81.5 ± 1.6	**0.001**	**<0.001**	**0.004**
Heart rate (b/min)	190 ± 4	182 ± 3	202 ± 4	0.19	**0.045**	**<0.001**
RSNA (nu)	6.4 ± 0.5	8.7 ± 0.6	9.6 ± 0.6	**0.012**	**0.001**	>0.5
RSNA amplitude (nu)	19.1 ± 1.2	23 ± 1.8	22.3 ± 1.2	0.20	>0.5	>0.5
RSNA frequency (bursts/s)	6.0 ± 0.2	7.3 ± 0.3	7.5 ± 0.2	**0.009**	**0.006**	>0.5
RSNA frequency (bursts/beat)	1.9 ± 0.1	2.4 ± 0.1	2.3 ± 0.1	**0.007**	0.081	>0.5
RSNA (μV)	18.3 ± 1.1	21.3 ± 0.9	24.8 ± 1.8	0.17	**0.002**	0.09
Nasopharyngeal (μV)	321 ± 37	298 ± 29	266 ± 17	>0.5	>0.5	>0.5
*Raw tissue weight*	*n* = 24	*n* = 28	*n* = 23			
Body weight (kg)	2.85 ± 0.05	2.85 ± 0.04	3.06 ± 0.05	>0.5	**0.004**	**0.002**
Total WAT (g)	49.6 ± 4.7	69.6 ± 4.8	81.9 ± 7.7	**0.028**	**<0.001**	0.27
Total WAT (% body weight)	1.7 ± 0.2	2.4 ± 0.2	2.6 ± 0.2	**0.013**	**0.002**	>0.5
*Estimates from DEXA*	*n* = 20	*n* = 14	*n* = 18			
Total Mass (kg)	2.86 ± 0.06	2.94 ± 0.08	3.10 ± 0.06	>0.5	**0.025**	0.26
Lean Muscle Mass (kg)	2.52 ± 0.05	2.51 ± 0.08	2.66 ± 0.04	>0.5	0.13	0.17
Fat Mass (g)	266 ± 20	352 ± 21	395 ± 29	**0.035**	**<0.001**	0.48
% Fat	9.5 ± 0.7	12.3 ± 0.7	12.9 ± 0.9	**0.031**	**0.003**	>0.5
*Biochemical measurements*	*n* = 5–6	*n* = 7–14	*n* = 5–9			
Plasma leptin (ng/ml)	2.14 ± 0.74	1.58 ± 0.39	2.58 ± 0.71	>0.5	>0.5	0.38
Glucose (mmol/l)	7.84 ± 0.22	8.13 ± 0.29	8.26 ± 0.48	>0.5	0.38	>0.5
Triglyceride (mmol/l)	0.52 ± 0.05	1.29 ± 0.59	2.1 ± 1.22	0.48	0.17	0.46
Cholesterol (mmol/l)	1.31 ± 0.33	1.45 ± 0.22	1.61 ± 0.39	>0.5	>0.5	>0.5
HDL cholesterol (mmol/l)	0.72 ± 0.12	0.71 ± 0.16	0.88 ± 0.22	>0.5	>0.5	0.48
LDL cholesterol (mmol/l)	0.66 ± 0.25	0.65 ± 0.14	0.74 ± 0.25	>0.5	>0.5	>0.5

Data are mean ± SEM. Probabilities for comparisons between dietary groups maternal control diet (mCD), maternal high-fat diet (mHFD), and mCD offspring fed a HFD for 10 days (mCD10d), shown in bold if *p* < 0.05. WAT, white adipose tissue; DEXA, dual-energy X-ray absorptiometry; MAP, mean arterial pressure; and nu, normalized units.

### Effect of Maternal Feeding on Cardiovascular Variables and RSNA in Offspring

Baseline MAP, RSNA, and HR in mCD rabbits were 69.4 ± 0.7 mmHg, 6.4 ± 0.5 nu, and 190 ± 4 b/min, respectively. In both mHFD and mCD10d rabbits, MAP was markedly greater than in mCD rabbits (75.8 mmHg, +9%; 81.5 mmHg, +17%, respectively, *P*_diet_ < 0.001; Table 2). MAP in mCD10d rabbits was 8% greater than mHFD rabbits (*P*_diet_ = 0.004). RSNA was markedly higher in mHFD (8.7 ± 0.6 nu, +35%) and mCD10d (9.6 ± 0.6 nu, +50%) compared to mCD rabbits (*P*_diet_ < 0.012) due mainly to greater burst frequency but not burst amplitude (Table 2). RSNA in mHFD and mCD10d rabbits was similar. HR was similar in mCD and mHFD groups but higher in mCD10d rabbits (*P*_diet_ < 0.001; Table 2).

### Effect of the LepR Antagonist on Cardiovascular Variables and RSNA in Offspring

**Figure 4:**
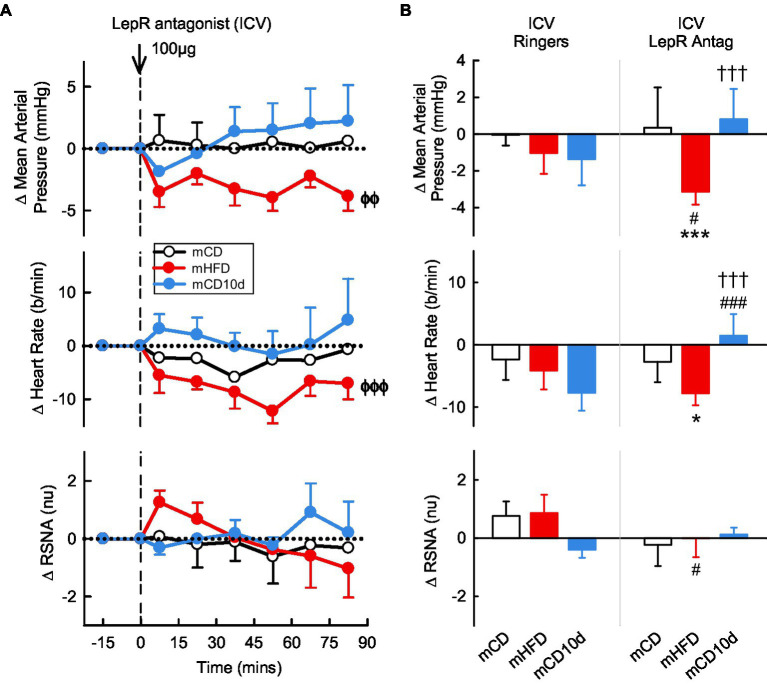
Effects of leptin-receptor (LepR) antagonist injected ICV. **(A)** Average changes in mean arterial pressure (MAP), heart rate, and RSNA (normalized units) from control in each group after ICV injection of LepR antagonist (100 μg, shown at dashed line) in maternal control diet (mCD; white, *n* = 7), maternal high fat diet (mHFD; red, *n* = 9), and mCD with 10d HFD (mCD10d; blue, *n* = 5) rabbits. Data are mean difference ± SED. ^ϕϕ^*p* < 0.01, ^ϕϕϕ^*p* < 0.001 for average effect of LepR antagonist. **(B)** Changes from control after both vehicle Ringer’s injection and LepR antagonist injection, averaged over all data points given ICV in mCD, mHFD, and mCD10d rabbits. Data are mean difference ± SED from the control period of each treatment group. ^*^p < 0.05 and ^***^p < 0.001 for comparison to mCD; ^†††^*p* < 0.001 for mHFD vs. mCD10d; and ^#^*p* < 0.05 and ^###^*p* < 0.001 for effect of LepR antagonist vs. Ringer’s.

In the mHFD group, we observed small but significant falls in MAP and HR after ICV administration of the LepR antagonist (*P*_drug_ < 0.003) but not RSNA (Figure 4). The hypotension was greater than in mCD and mCD10d groups in which the LepR antagonist had little effect on any variable, and it was also greater than the effect of vehicle (*P*_veh_ = 0.03; Figure 4). The bradycardia produced by ICV LepR antagonist in the mHFD rabbits was greater than the response in mCD10d (*P*_diet_ < 0.001) and mCD rabbits (*P*_diet_ = 0.03). In mCD10d rabbits, the effect on HR was different to that after vehicle (*P*_veh_ < 0.001; Figure 4).

**Figure 5:**
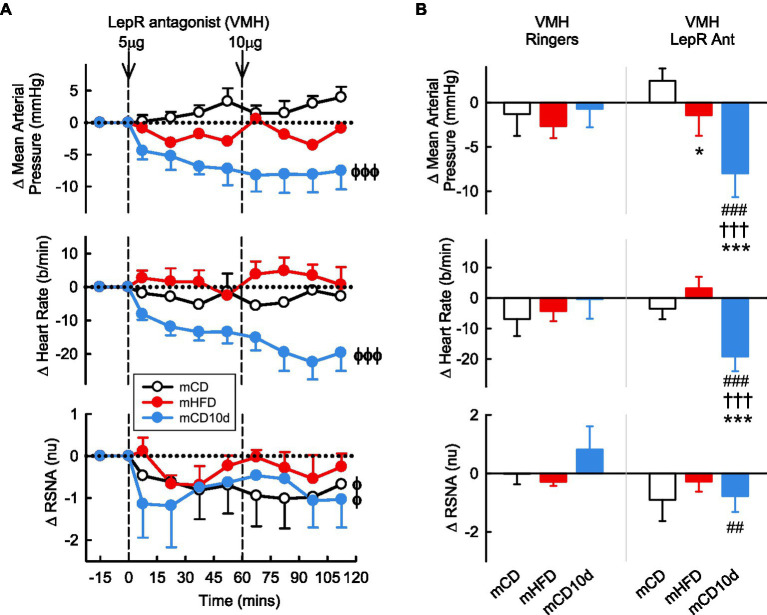
Effects of LepR antagonist injected into the VMH. **(A)** Average changes in MAP, heart rate, and RSNA (normalized units) from control in each group after VMH injection of LepR antagonist (5, 10 μg, shown at dashed lines) in mCD (white, *n* = 6), mHFD (red, *n* = 6), and mCD10d (blue, *n* = 6) rabbits. Data are mean difference ± SED. ^ϕ^*p* < 0.05, ^ϕϕϕ^*p* < 0.001 for average effect of LepR antagonist. **(B)** Changes from control after administration of vehicle Ringer’s and LepR antagonist, averaged over the second dose, given into the VMH in mCD, mHFD, and mCD10d rabbits. Data are mean difference ± SED from the control period of each treatment group. ^*^p < 0.05 and ^***^p < 0.001 for comparison to mCD; ^†††^*p* < 0.001 for mHFD vs. mCD10d; ^##^*p* < 0.01 and ^###^*p* < 0.001 for effect of LepR antagonist vs. Ringer’s.

VMH administration of the LepR antagonist induced marked falls in MAP (−8.0 ± 3.0 mmHg, −9%,*P*_drug_ < 0.001) and HR (−19 ± 5 b/min, −9%, *P*_drug_ < 0.001) in the mCD10d group compared to control (Figure 5) but had no effect in the other groups. Thus, the changes in MAP and HR in mCD10d offspring were greater compared to the other groups (both *P*_diet_ < 0.001) and were also greater than the small changes observed after vehicle in that group (*P*_veh_ < 0.001; Figure 5). VMH administration of the LepR antagonist also reduced RSNA (−0.8 ± 0.5 nu, −7%, *P*_drug_ = 0.01) in the mCD10d group, but this reduction in RSNA was not different to the non-significant effects in mCD or mHFD rabbit although it was different to the small increase after vehicle injection (*P*_veh_ = 0.002; Figure 5).

### Effect of αMSH on Cardiovascular Variables and RSNA in Offspring

**Figure 6:**
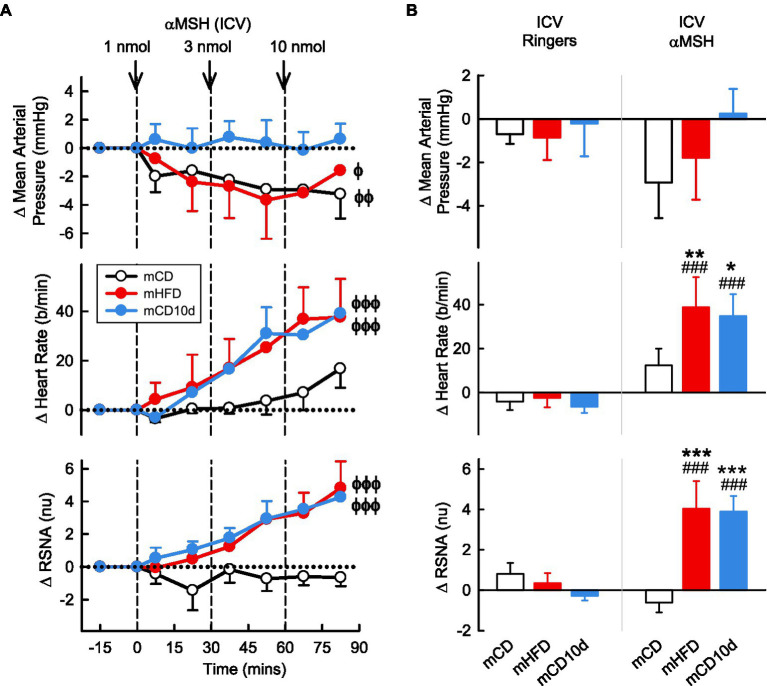
Effects of alpha-melanocyte-stimulating hormone (αMSH) injected ICV. **(A)** Average changes in MAP, heart rate, and RSNA (normalized units) from control in each group after ICV injection of αMSH (1, 3, and 10 nmol, shown at dashed lines) in mCD (white, *n* = 9), mHFD (red, *n* = 8), and mCD10d (blue, *n* = 6) rabbits. Data are mean difference ± SED. ^ϕ^*p* < 0.05, ^ϕϕ^*p* < 0.01 and ^ϕϕϕ^*p* < 0.001 for average effect of αMSH. **(B)** Changes from control after administration of vehicle Ringer’s and αMSH, averaged over the third dose, given ICV in mCD, mHFD, and mCD10d rabbits. Data are mean difference ± SED from the control period of each treatment group. ^*^p < 0.05, ^**^p < 0.01, and ^***^p < 0.001 for comparison to mCD; ^###^*p* < 0.001 for effect of αMSH vs. Ringer’s.

ICV administration of αMSH had little effect on MAP in the mCD10d group but reduced MAP slightly in the mHFD and mCD groups (*P*_drug_ = 0.01; Figure 6). By contrast, ICV administration of αMSH resulted in marked dose dependent increases in RSNA in mHFD and mCD10d rabbits (+4.0 ± 1.4 nu, +45% and +3.9 ± 0.8 nu, +51%, respectively, *P*_drug_ < 0.001), but no effect on the RSNA in mCD rabbits (Figure 6). HR also increased in the mHFD and mCD10d rabbits (+39 ± 14 b/min, +23% and +35 ± 10 b/min, +19%, respectively, *P*_drug_ < 0.001; Figure 6). The increases in RSNA and HR were greater in mHFD and mCD10d rabbits than in mCD (all *P*_diet_ < 0.02) and were also greater than after vehicle treatment (all *P*_veh_ < 0.001; Figure 6).

**Figure 7:**
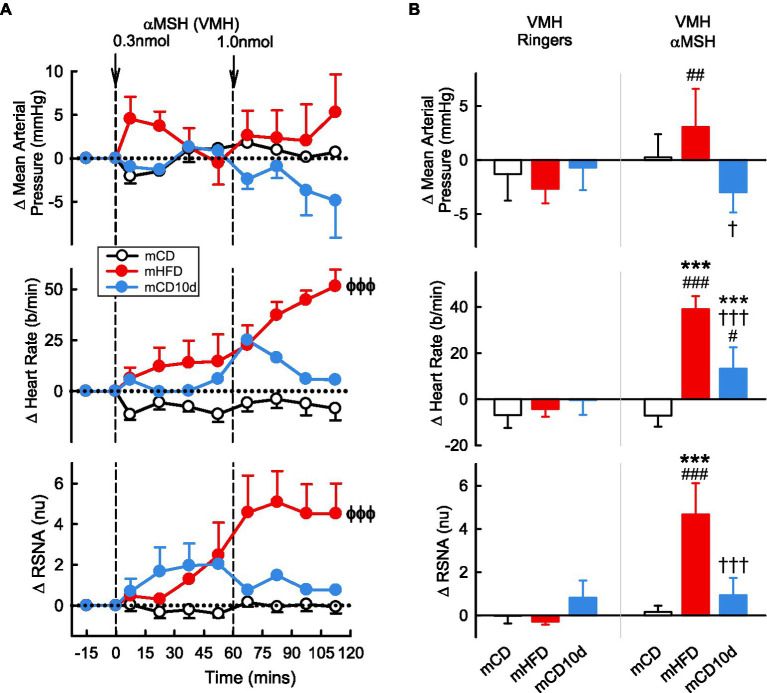
Effects of αMSH injected in the VMH. **(A)** Average changes in MAP, heart rate, and RSNA (normalized units) from control in each group after VMH injection of αMSH (0.3, 1.0 nmol, shown at dashed lines) in mCD (white, *n* = 8), mHFD (red, *n* = 7), and mCD10d (blue, *n* = 4) rabbits. Data are mean difference ± SED. ^ϕϕϕ^*p* < 0.001 for average effect of αMSH. **(B)** Changes from control after administration of vehicle Ringer’s and αMSH, averaged over the second dose, given into the VMH in mCD, mHFD, and mCD10d rabbits. Data are mean difference ± SED from the control period of each treatment group. ^***^p < 0.001 for comparison to mCD, ^†^*p* < 0.05 and ^†††^*p* < 0.001 for mHFD vs. mCD10d; and ^##^*p* < 0.01 and ^###^*p* < 0.001 for effect of αMSH vs. Ringer’s.

VMH administration of αMSH increased RSNA and HR compared to control but only in mHFD rabbits (+4.7 ± 1.5 nu, +85%; +39 ± 6 b/min, +23%, *P*_drug_ < 0.001; Figure 7). Thus, the effect on HR was greater in mHFD than both mCD and mCD10d rabbits (*P*_diet_ < 0.001) but in mCD10d rabbits, there was a small increase in HR after the second dose which was greater than in the mCD group (+13 ± 9 b/min, 7%, *P*_diet_ < 0.001). The effect on RSNA was greater in mHFD than mCD (*P*_diet_ < 0.001), and both RSNA and HR increases were greater than the effect of vehicle (*P*_veh_ < 0.001; Figure 7).

### Effect of SHU9119 on Cardiovascular Variables and RSNA in Offspring

**Figure 8:**
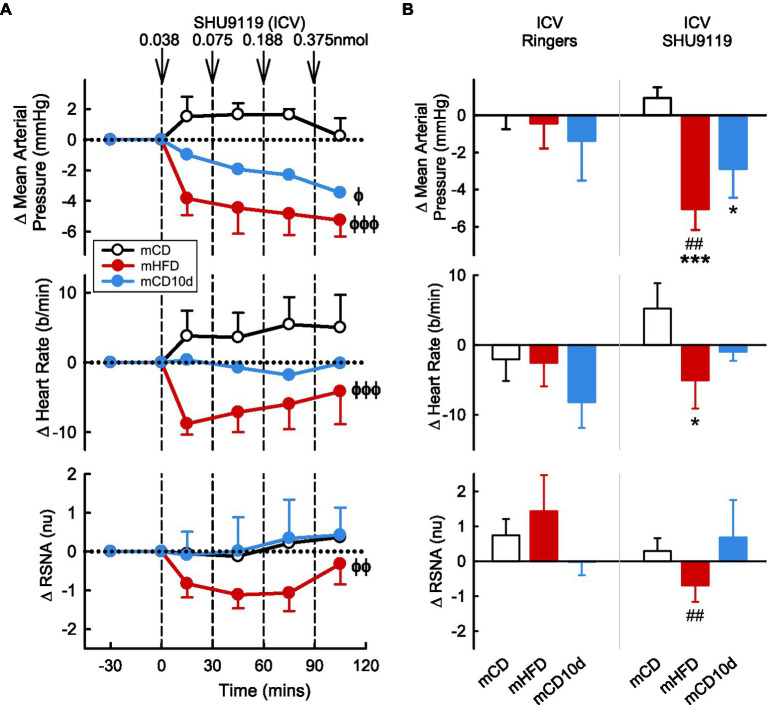
Effects of SHU9119 injected ICV. **(A)** Average changes in MAP, heart rate, and RSNA (normalized units) from control in each group after ICV administration of SHU9119 (0.038, 0.075, 0.188, and 0.375 nmol, shown at dashed lines) in mCD (white, *n* = 8), mHFD (red, *n* = 8), and mCD10d (blue, *n* = 6) rabbits. Data are mean difference ± SED. ^ϕ^*p* < 0.05, ^ϕϕ^*p* < 0.01, ^ϕϕϕ^*p* < 0.001 for average effect of SHU9119. **(B)** Changes from control after administration of vehicle Ringer’s and SHU9119, averaged over the last two doses, given ICV in mCD, mHFD, and mCD10d rabbits. Data are mean difference ± SED from the control period of each treatment group. ^*^p < 0.05, ^***^p < 0.001 for comparison to mCD; ^##^*p* < 0.01 for effect of SHU9119 vs. Ringer’s.

ICV administration of the MC3/4-receptor antagonist SHU9119 reduced MAP, HR, and RSNA in mHFD (−5.1 ± 1.1 mmHg *P*_drug_ < 0.001, −5 ± 4 b/min *P*_drug_ < 0.001, −0.7 ± 0.5 nu *P*_drug_ = 0.005, respectively) but had no effect in mCD (*P*_diet_ < 0.05; Figure 8). The falls in MAP and RSNA were greater than those observed after vehicle (*P*_veh_ < 0.003). In the mCD10d group, ICV SHU9119 also reduced MAP (−2.9 ± 1.6 mmHg, *P*_drug_ = 0.02) but had no effect on HR or RSNA (Figure 8).

**Figure 9:**
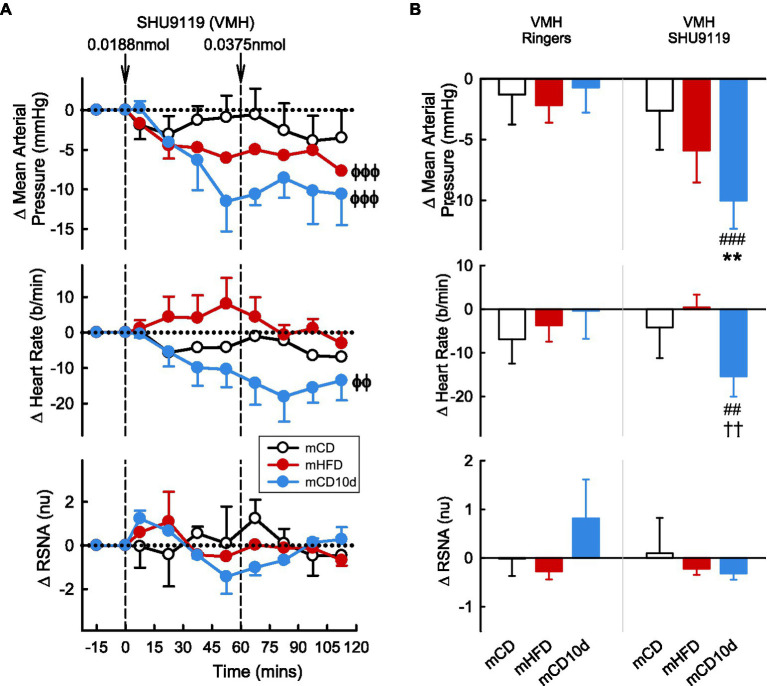
Effects of SHU9119 injected in the VMH. **(A)** Average changes MAP, heart rate, and RSNA (normalized units) from control in each group after VMH injection of SHU9119 (0.0188 nmol, 0.0375 nmol) in mCD (white, *n* = 5), mHFD (red, *n* = 6), and mCD10d (blue, *n* = 3) rabbits. Data are mean difference ± SED. ^ϕϕ^*p* < 0.01, ^ϕϕϕ^*p* < 0.001 for average effect of SHU9119. **(B)** Changes from control after administration of vehicle Ringer’s and SHU9119, averaged over second dose, given into the VMH in mCD, mHFD, and mCD10d rabbits. Data are mean difference ± SED from the control period of each treatment group. ^**^p < 0.01 for comparison to mCD; ^††^*p* < 0.01 for mHFD vs. mCD10d; and ^##^*p* < 0.01 and ^###^*p* < 0.001 for effect of SHU9119 vs. Ringer’s.

VMH administration of SHU9119 markedly reduced MAP in mHFD (−5.9 ± 2.6 mmHg, −8%, *P*_drug_ < 0.001) and in mCD10d rabbits (−10.0 ± 2.3 mmHg, −13%, *P*_drug_ < 0.001) but had no effect in the mCD group (Figure 9). Treatment also reduced HR in the mCD10d group (−15 ± 5 b/min, −8%, *P*_drug_ = 0.01) but not in others, and these effects were significantly greater than those observed after vehicle (*P*_veh_ < 0.01; Figure 9). VMH administration of SHU9119 did not affect RSNA in any group (Figure 9).

### Effect of InsR Antagonist on Cardiovascular Variables and RSNA in Offspring

**Figure 10:**
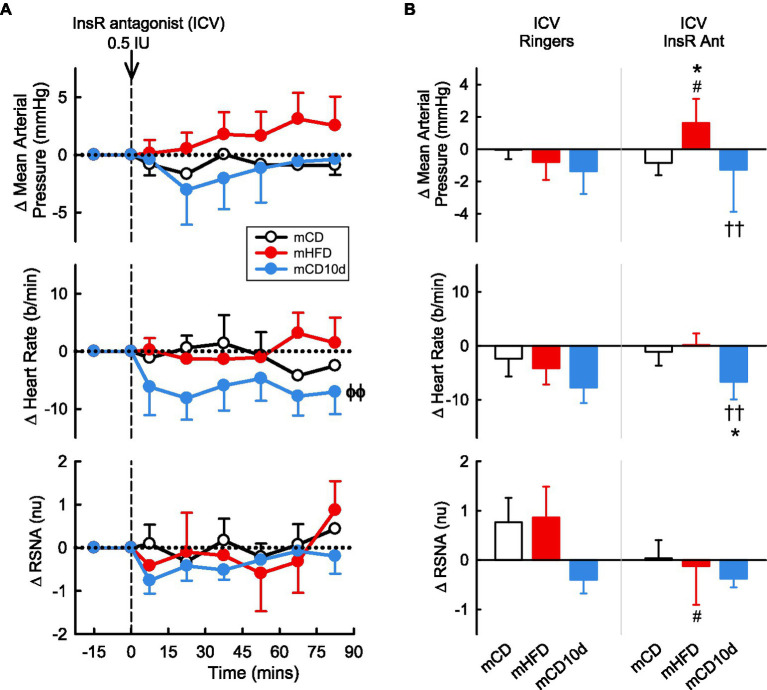
Effects of insulin-receptor (InsR) antagonist injected ICV. **(A)** Average changes in MAP, heart rate, and RSNA (normalized units) from control in each group after ICV injection of InsR antagonist (0.5 IU, shown at dashed line) in mCD (white, *n* = 6), mHFD (red, *n* = 9), and mCD10d (blue, *n* = 6) rabbits. Data are mean difference ± SED. ^ϕϕ^*p* < 0.05 for average effect of InsR antagonist. **(B)** Changes from control after administration of vehicle Ringer’s and InsR antagonist, averaged over all data points, given ICV in mCD, mHFD, and mCD10d rabbits. Data are mean difference ± SED from the control period of each treatment group. ^*^p < 0.05 for comparison to mCD; ^††^*p* < 0.01 for mHFD vs. mCD10d; and ^#^*p* < 0.05 for effect of InsR antagonist vs. Ringer’s.

**Figure 11:**
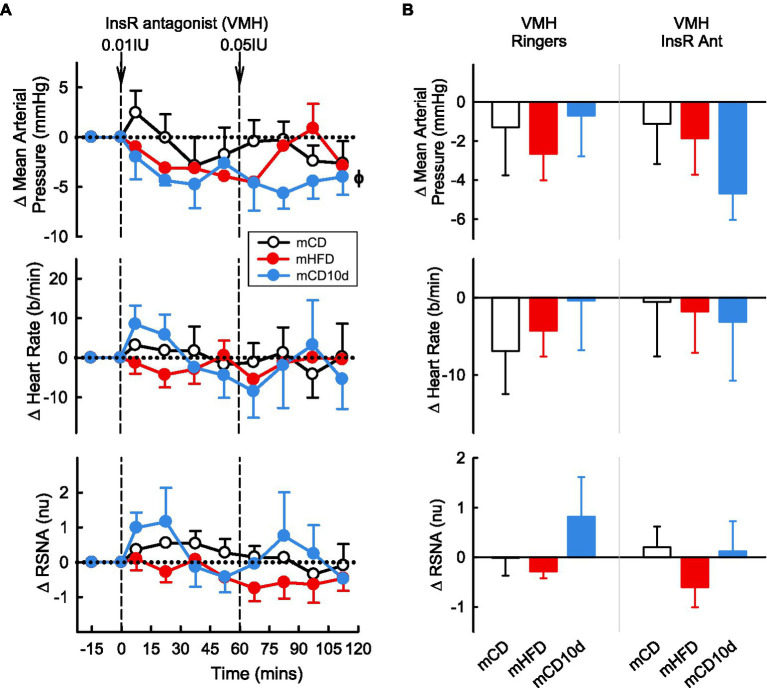
Effects of InsR antagonist injected in the VMH. **(A)** Average changes in MAP, heart rate, and RSNA (normalized units) from control in each group after VMH injection of InsR antagonist (0.01, 0.05 IU, shown at dashed lines) in mCD (white, *n* = 7), mHFD (red, *n* = 5), and mCD10d (blue, *n* = 3) rabbits. Data are mean difference ± SED. ^ϕ^*p* < 0.05 for average effect of InsR antagonist. **(B)** Changes from control after administration of vehicle Ringer’s and InsR antagonist, averaged over the second dose, given into the VMH in mCD, mHFD, and mCD10d rabbits. Data are mean difference ± SED from the control period of each treatment group.

A 0.5 IU dose of InsR antagonist administered ICV or 0.01 and 0.05 IU doses given directly into the VMH had mostly no observable effect on any parameter in any group (Figures 10, 11). In mCD10d offspring, there was a small reduction in HR (−7 ± 3 b/min *P*_drug_ = 0.004) following ICV administration while after VMH administration there was a small fall in MAP (−4.7 ± 1.4 mmHg *P*_drug_ = 0.03) but this was not different to effects in the other groups (Figure 11).

### Effect of Vehicle on Cardiovascular Variables and RSNA in Offspring

**Figure 12:**
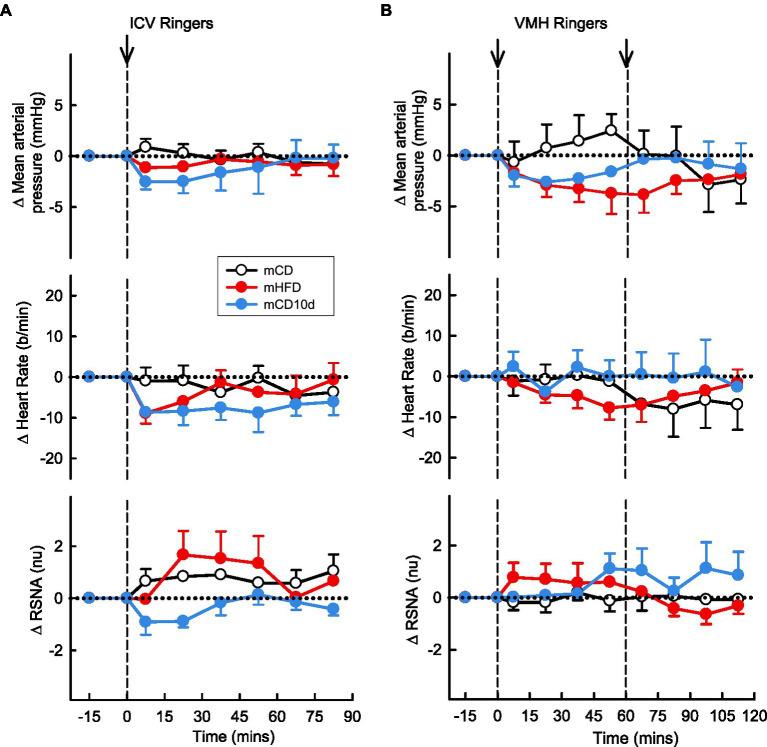
Effects of Ringer’s solution injected ICV and into the VMH. Average changes in MAP, heart rate, and RSNA (normalized units) from control in each group after **(A)**: ICV Ringer’s (vehicle) and **(B)**: VMH administration of Ringer’s (vehicle) in mCD [white, **(A)** *n* = 9, **(B)** *n* = 5], mHFD [red, **(A)** *n* = 8, **(B)** *n* = 9], and mCD10d [blue, **(A)** *n* = 8, **(B)** *n* = 7] rabbits. Data are mean difference ± SED.

The vehicle Ringer’s solution injected ICV and into the VMH had no observable effects on MAP, RSNA, or HR in any group (Figure 12).

### Effects of Maternal and Adult HFD on mRNA Expression in the Hypothalamus of Offspring

**Figure 13:**
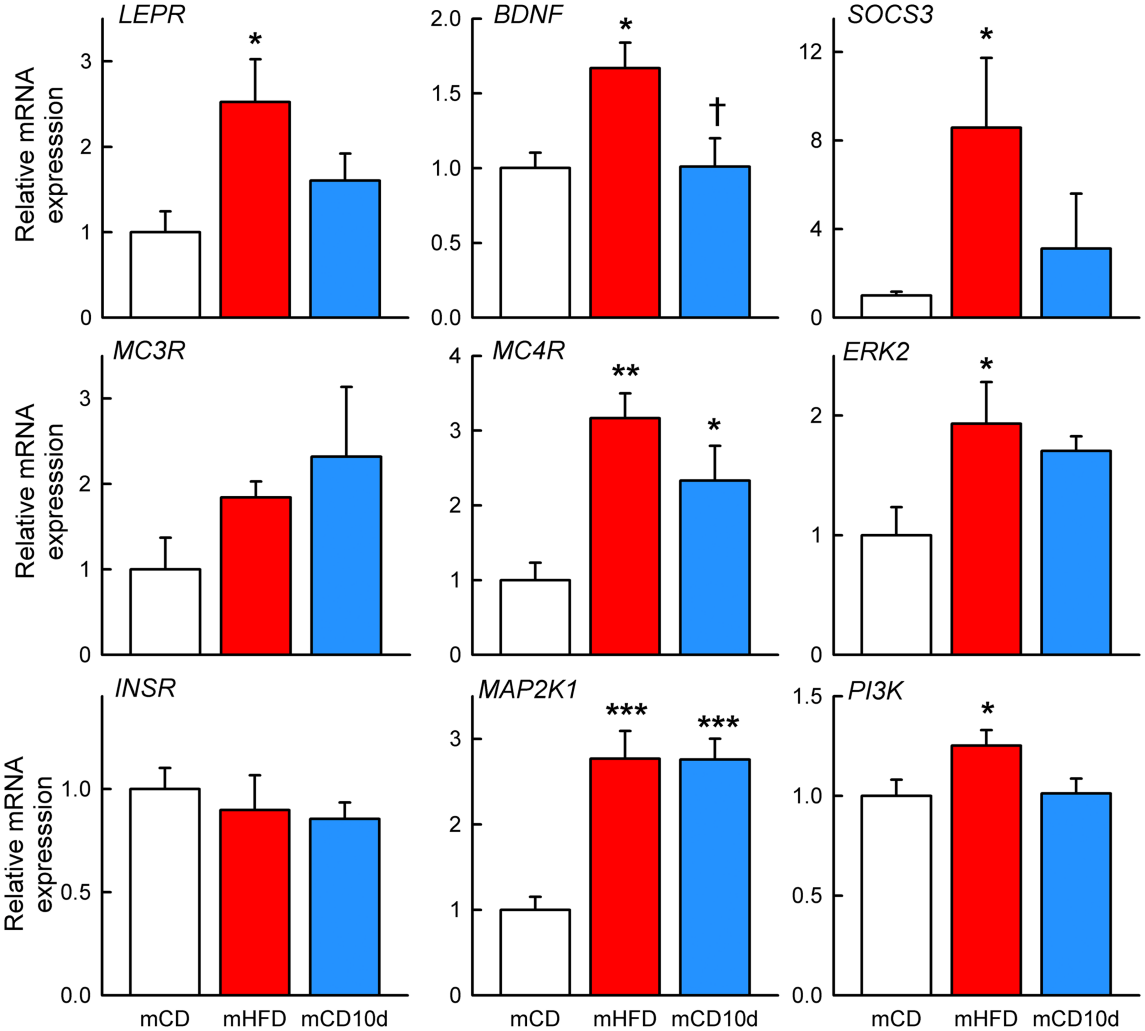
Hypothalamic mRNA expression in offspring. Relative expression of mRNA in mHFD (red bars, *n* = 4–5) and mCD10d (blue bars, *n* = 5–6) rabbits compared to mCD (white bars, *n* = 4–5). Relative expression of *LEPR*, *BDNF*, suppressor of cytokine signaling 3, melanocortin 3 and 4 receptors, extracellular signal-regulated kinase-2, InsR, mitogen-activated protein kinase kinase 1, and phosphatidylinositol-3-kinase. Data are mean ± SEM. Open circles represent individual data points. ^*^p < 0.05, ^**^p < 0.01, and ^***^p < 0.001 for comparison to the mCD group. ^†^*p* < 0.05 for comparison to the mHFD group.

mHFD rabbits showed greater hypothalamic mRNA expression of *LEPR* (+153%, *P*_diet_ = 0.013), *BDNF* (+67%, *P*_diet_ = 0.012), *SOCS3* (+757%, *P*_diet_ = 0.043), *MC4R* (+217%, *P*_diet_ = 0.001), *ERK2* (+93%, *P*_diet_ = 0.020), *MAP2K1* (+177%, *P*_diet_ < 0.001), and *PI3K* (+25%, *P*_diet_ = 0.045) when compared to mCD rabbits (Figure 13). By contrast, mRNA expression was only higher in mCD10d than in mCD for *MC4R* (+133%, *P*_diet_ = 0.022) and *MAP2K1* (+176%, *P*_diet_ < 0.001) with other mRNA expression levels similar to mCD (Figure 13). *BDNF* mRNA expression levels were 65% higher in mHFD rabbits than in mCD10d rabbits (*P*_diet_ = 0.013; Figure 13). There was no difference in the mRNA expression of *MC3R* and *INSR* among any of the groups.

## Discussion

In the present study, we examined central leptin-melanocortin and insulin signaling pathways mediating the higher MAP and RSNA in the adult offspring of rabbits fed a HFD during pregnancy and lactation. Since there were residual fat deposits in the mHFD offspring that may mediate some of the differences we observe between these animals and control mCD, we also fed a group of mCD rabbits a HFD for a short period of 10 days and then allowed a 3-week recovery period on a chow diet. This recovery period allowed for any acute consequences of HFD consumption to stabilize. Our main findings were that the higher MAP in mHFD and mCD10d offspring was partly due to greater central leptin signaling and associated greater receptor expression but likely at distinct sites within the hypothalamus. The LepR antagonist had the most effect in lowering MAP and HR when injected into the VMH of mCD10d offspring, but in mHFD rabbits, the hypotensive effect was only observed ICV. These effects are not likely to involve renal SNA as no effects were observed on this parameter by the LepR antagonist. The size of the effects on MAP and HR was small, and the results should thus be interpreted carefully. The enhanced melanocortin contribution to the hypertension as detected by SHU9119 was common to both groups suggesting that residual fat rather than maternal programming was responsible. However, the sympatho-inhibition by ICV SHU9119 and the sympatho-excitation by αMSH only occurred in mHFD suggesting the mechanism involved maternal HFD programming. Interestingly, SHU9119 was effective when administered ICV and less so within the VMH suggesting that the tonic activation of the melanocortin system occurred within another region or nucleus. By contrast, the marked sympatho-excitation produced by αMSH only in mHFD occurred within the VMH and also when given ICV. We also observed a distinct pattern of increased expression of hypothalamic signaling molecules in the mHFD group compared to mCD and particularly marked for LepR, BDNF, and SOCS3. These are likely due to maternal programming rather than the effects of a short period of HFD in adulthood. Taken together, these findings suggest hypertension and sympatho-excitation may occur through a manifest alteration to central leptin-melanocortin signaling pathways in the hypothalamus due mainly to maternal programming but also to a significant contribution from residual visceral fat.

### Developmental Programming of Neuropeptide and Adipokine Receptor Signaling

Developmental programming by maternal obesity during pregnancy and during growth is mediated by long-term changes in expression of peptides and adipokine receptors, such as for leptin in the hypothalamic circuits (Bouret et al., 2004). Leptin is secreted primarily by adipocytes and is thought to be present in direct proportion to the percentage of adipose tissue. This contrasts the current study and our previous studies of adult rabbits which were fed a HFD but then returned to a CD where plasma levels of leptin were in the normal range despite the presence of substantial visceral fat (Armitage et al., 2012). Importantly, MAP and RSNA remained elevated compared to control animals. We have previously shown that the offspring (fed a CD from weaning) from mothers fed a HFD during pregnancy and lactation also have high MAP and RSNA as well as a markedly increased pressor and RSNA sensitivity to ICV leptin (Prior et al., 2014). This suggests that leptin can reprogram central melanocortin pathways during development and also in adulthood. It is well known that during the critical period in brain development, leptin plays a permissive neurotrophic role, establishing the neural circuitry of the hypothalamus *via* the growth of axons and neuronal differentiation and migration (Bouret et al., 2004). Leptin acts at the long form of the Ob receptor (also known as LepR) found in the lateral hypothalamic area, hypothalamic arcuate, VMH, and paraventricular nucleus (Elmquist, 2001). Activation of the LepR stimulates intracellular signaling pathways, including those mediated by signal transducer and activator of transcription 3 *(STAT3), PI3K*, and *ERK 1 or 2* (Myers et al., 2008). Leptin-receptor driven activation of *STAT3* is particularly important for development of arcuate projections and architecture of pro-opiomelanocortin (POMC) but not neuropeptide Y (NPY) projections (Bouret et al., 2012). Suppressor of cytokine signaling 3 is a leptin target gene that prevents over-activation of the leptin signaling pathway (Bjorbaek et al., 1998), and in general, leptin can induce a negative feedback through *SOCS3* accumulation during prolonged LepR stimulation (Banks et al., 2000; Bates et al., 2004). Thus, in the current study, it was important to separate the developmental programming from the acute effect of elevated body fat in adulthood or by changes in circulating lipids which we did by incorporating a group that had a short 10-days of HFD followed by 3-weeks of CD. In this way, we can see that the higher levels of *LEPR* and *SOCS3* mRNA expression were only observed in the mHFD group which indicates that there was higher intracellular activity in the leptin signaling pathway. Importantly, only in this group, the LepR antagonist given ICV had a small effect in lowering MAP but did not affect RSNA suggesting that the maternal programming may have had an impact in increasing MAP but through non-renal sympathetic vasomotor tone. The antagonist was ineffective in mHFD when given into the VMH suggesting that another area of the hypothalamus might be the site of action. We speculate that this early life programming contribution to the hypertension might be related to arcuate neurons and their projections to other regions of the hypothalamus, such as the dorsomedial hypothalamus (Simonds et al., 2014) or paraventricular nucleus (da Silva et al., 2014).

A major target of circulating leptin in the arcuate is POMC neurons that release αMSH and also contain cocaine-and-amphetamine-related transcript (Elmquist, 2001). POMC and NPY neurons in the arcuate project to many regions of the hypothalamus and beyond to influence appetite and metabolism (Cowley et al., 2001; da Silva et al., 2014). We have previously investigated the contribution of NPY and POMC systems in obesity-induced hypertension and activation of RSNA in adult rabbits on a 3-week HFD (Barzel et al., 2016). We observed a marked hypotensive and sympatho-inhibitory effect of SHU9119 (a MC3/4 receptor antagonist) but no effect of an NPY receptor antagonist (Barzel et al., 2016). In the current study, we found similar hypotensive effects of SHU9119 when injected either ICV or into the VMH in both the mHFD and mCD10d rabbits suggesting that blocking a tonic melanocortin pressor action within the VMH was likely due to the presence of visceral fat rather than due to maternal programming. However, we did observe a reduction in RSNA with ICV SHU9119 in mHFD rabbits that was not observed in mCD10d rabbits. This implies that the renal vasomotor pathways are differentially affected by maternal programming. Indeed, the marked sympatho-excitatory action of VMH αMSH in mHFD rabbits was not present in the mCD10d rabbits either.

### Programming of Neuronal Plasticity: Role of BDNF

We have shown that higher renal SNA is due to amplification of melanocortin-receptor signaling within the VMH during a 3-week HFD in adult rabbits and is associated with activation of BDNF and upregulation of MC4R. Importantly, in the current study, we also saw selective activation of BDNF as well as a more marked activation of MC4 receptors in the hypothalamus of mHFD rabbits compared to mCD and mCD10d groups. BDNF is a well-known neurotrophin which regulates neuronal development, supports differentiation, maturation, and survival of the neurons and importantly, modulates synaptic plasticity (Huang and Reichardt, 2001; Binder and Scharfman, 2004). The plasticity of the brain refers to the natural capacity of the brain to modify its structure and function through experience. At the level of synapses, plasticity includes functional changes that strengthen or weaken existing synapses (Ellacott and Cone, 2006). BDNF promotes stability of dendritic synapses of the hypothalamic neurons, and it has been shown that neurodevelopmental anomalies involving BDNF contribute to the obesity phenotype (Rios et al., 2001). Increased release of αMSH in the VMH and activation of MC4R is known to induce increased signaling related to other functions (Toda et al., 2013) and involves activation of mitogen-activated protein kinase (MEK) and ERK pathway. This activates transcriptional factor cAMP-responsive element-binding protein and the transcription of plasticity-associated genes and synthesis of secretory BDNF (Caruso et al., 2014). Indeed, in the current study, we observed increases in both *MAP2K1* and *ERK2* mRNA expression in the hypothalamus of mHFD offspring, evidence that these activated intermediaries, as part of this pathway, are involved in the maternal programming by a HFD of the adult offspring and that the mechanism involves neuronal plasticity. However, we did also see increased expression in the mCD10d group but this was not associated with BDNF. In support of a role of BDNF in mediating the maternal programming and also the effect of a HFD in adulthood to upregulate melanocortin signaling, we know that a MEK inhibitor can prevent leptin-induced enhanced MC3/4 receptor signaling in the VMH (Toda et al., 2013). Acute administration of glucose increases BDNF expression in the VMH and mice with depleted BDNF in the VMH and dorsomedial hypothalamus become obese (Unger et al., 2007). BDNF is highly expressed in the VMH and mice with mutant MC4R have low levels of tropomyosin receptor kinase B (the BDNF receptor; Xu et al., 2003). Central infusion of a melanocortin agonist upregulates BDNF in the VMH (Xu et al., 2003).

### Maternal Obesity and Insulin Signaling Pathway

Offspring from obese mothers display reduced activity of the insulin signaling pathway as well as insulin-receptor mRNA expression (Yan et al., 2010). In the current study, neither the mHFD nor the mCD10d offspring group responded with changes in MAP or RSNA when the InsR antagonist was administered ICV or into the VMH suggesting little role of insulin in mediating the hypertension or sympatho-excitation. In addition, InsR expression in the hypothalamus of mHFD rabbits was not different to any other groups. We did observe an increase in *PI3K* expression in the hypothalamus of mHFD rabbits, and insulin is known to activate PI3K signaling (Hirsch et al., 2007) influencing cell proliferation, metabolism, cell growth, and intracellular trafficking (Engelman et al., 2006). The increase in *PI3K* expression but not *INSR* expression suggests evidence of insulin resistance and/or an altered insulin signaling pathway in the hypothalamus of this group.

We conclude that the higher MAP in mHFD and mCD10d offspring is likely due to greater central leptin signaling at distinct sites within the hypothalamus while the enhanced melanocortin contribution, as determined by SHU9119, was common to both groups suggesting that residual body fat is likely to be the cause. However, the effects of SHU9119 and αMSH on RSNA pathways only in mHFD suggest a maternal HFD may program sympatho-excitatory capacity in these offspring and that this may involve increased leptin receptor and BDNF expression. The programmed hyper sympathetic responsivity is therefore primed to be activated by a HFD in adulthood which would likely produce a much greater hypertension than otherwise would be expected. However, this needs to be tested experimentally in future experiments to confirm such a scenario and also to determine whether BDNF is the underlying neurotrophic factor responsible.

## Data Availability Statement

The raw data supporting the conclusions of this article will be made available by the authors, without undue reservation.

## Ethics Statement

The animal study was reviewed and approved by Alfred Medical Research Education Precinct Animal Ethics Committee.

## Disclosure

GH has received research support from the Boehringer Ingelheim for studies unrelated to the current study.

## Author Contributions

GH, JA, and KL designed the study. GH, KL, SB, FM, KJ, CG, YS, and JA performed the study including experimental data collection and analysis, preparation, and writing and editing of the manuscript. All authors contributed to the article and approved the submitted version.

### Conflict of Interest

GH has received research support from the Boehringer Ingelheim for studies unrelated to the current study.

The remaining authors declare that the research was conducted in the absence of any commercial or financial relationships that could be construed as a potential conflict of interest.

## References

[ref1] Armitage J. A. Burke S. L. Prior L. J. Barzel B. Eikelis N. Lim K.. (2012). Rapid onset of renal sympathetic nerve activation in rabbits fed a high-fat diet. Hypertension 60, 163–171. 10.1161/HYPERTENSIONAHA.111.190413, PMID: 22647890

[ref2] Banks A. S. Davis S. M. Bates S. H. Myers M. G. Jr. (2000). Activation of downstream signals by the long form of the leptin receptor. J. Biol. Chem. 275, 14563–14572. 10.1074/jbc.275.19.14563, PMID: 10799542

[ref3] Barzel B. Lim K. Davern P. J. Burke S. L. Armitage J. A. Head G. A. (2016). Central pro-opiomelanocortin but not neuropeptide Y mediates sympatho-excitation and hypertension in fat fed conscious rabbits. J. Hypertens. 34, 464–473. 10.1097/HJH.0000000000000811, PMID: 26820476

[ref4] Bates S. H. Dundon T. A. Seifert M. Carlson M. Maratos-Flier E. Myers M. G. Jr. (2004). LRb-STAT3 signaling is required for the neuroendocrine regulation of energy expenditure by leptin. Diabetes 53, 3067–3073. 10.2337/diabetes.53.12.3067, PMID: 15561935

[ref5] Binder D. K. Scharfman H. E. (2004). Brain-derived neurotrophic factor. Growth Factors 22, 123–131. 10.1080/08977190410001723308 15518235 PMC2504526

[ref6] Bjorbaek C. Elmquist J. K. Frantz J. D. Shoelson S. E. Flier J. S. (1998). Identification of SOCS-3 as a potential mediator of central leptin resistance. Mol. Cell 1, 619–625. 10.1016/S1097-2765(00)80062-3, PMID: 9660946

[ref7] Bluher M. (2019). Obesity: global epidemiology and pathogenesis. Nat. Rev. Endocrinol. 15, 288–298. 10.1038/s41574-019-0176-8, PMID: 30814686

[ref8] Bouret S. G. Bates S. H. Chen S. Myers M. G. Jr. Simerly R. B. (2012). Distinct roles for specific leptin receptor signals in the development of hypothalamic feeding circuits. J. Neurosci. 32, 1244–1252. 10.1523/JNEUROSCI.2277-11.2012, PMID: 22279209 PMC3567460

[ref9] Bouret S. G. Draper S. J. Simerly R. B. (2004). Trophic action of leptin on hypothalamic neurons that regulate feeding. Science 304, 108–110. 10.1126/science.1095004, PMID: 15064420

[ref10] Bringhenti I. Ornellas F. Martins M. A. Mandarim-de-Lacerda C. A. Aguila M. B. (2015). Early hepatic insult in the offspring of obese maternal mice. Nutr. Res. 35, 136–145. 10.1016/j.nutres.2014.11.006, PMID: 25582085

[ref11] Burguera B. Couce M. E. (2001). Leptin access into the brain. A saturated transport mechanism in obesity. Physiol. Behav. 74, 717–720. 10.1016/S0031-9384(01)00615-1, PMID: 11790434

[ref12] Burke S. L. Head G. A. (2003). Method for in vivo calibration of renal sympathetic nerve activity in rabbits. J. Neurosci. Methods 127, 63–74. 10.1016/S0165-0270(03)00121-3 12865149

[ref13] Burke S. L. Lim K. Moretti J. L. Head G. A. (2016). Comparison of sympathetic nerve activity normalization procedures in conscious rabbits. Am. J. Physiol. Heart Circ. Physiol. 310, H1222–H1232. 10.1152/ajpheart.00866.2015 26921439

[ref14] Caruso V. Lagerstrom M. C. Olszewski P. K. Fredriksson R. Schioth H. B. (2014). Synaptic changes induced by melanocortin signalling. Nat. Rev. Neurosci. 15, 98–110. 10.1038/nrn3657, PMID: 24588018

[ref15] Cowley M. A. Smart J. L. Rubinstein M. Cerdan M. G. Diano S. Horvath T. L.. (2001). Leptin activates anorexigenic POMC neurons through a neural network in the arcuate nucleus. Nature 411, 480–484. 10.1038/35078085, PMID: 11373681

[ref16] da Silva A. A. do Carmo J. M. Wang Z. Hall J. E. (2014). The brain Melanocortin system, sympathetic control, and obesity hypertension. Physiology 29, 196–202. 10.1152/physiol.00061.2013, PMID: 24789984 PMC4046815

[ref17] Desai M. Beall M. Ross M. G. (2013). Developmental origins of obesity: programmed adipogenesis. Curr. Diab. Rep. 13, 27–33. 10.1007/s11892-012-0344-x, PMID: 23188593 PMC3563293

[ref18] Dhillon H. Kalra S. P. Prima V. Zolotukhin S. Scarpace P. J. Moldawer L. L.. (2001). Central leptin gene therapy suppresses body weight gain, adiposity and serum insulin without affecting food consumption in normal rats: a long-term study. Regul. Pept. 99, 69–77. 10.1016/S0167-0115(01)00237-3, PMID: 11384767

[ref19] Dorward P. K. Riedel W. Burke S. L. Gipps J. Korner P. I. (1985). The renal sympathetic baroreflex in the rabbit. Arterial and cardiac baroreceptor influences, resetting, and effect of anesthesia. Circ. Res. 57, 618–633. 10.1161/01.RES.57.4.618, PMID: 4042286

[ref20] Ellacott K. L. Cone R. D. (2006). The role of the central melanocortin system in the regulation of food intake and energy homeostasis: lessons from mouse models. Philos. Trans. R. Soc. B: Biol. Sci. 361, 1265–1274. 10.1098/rstb.2006.1861, PMID: 16815803 PMC1642695

[ref21] Elmquist J. K. (2001). Hypothalamic pathways underlying the endocrine, autonomic, and behavioral effects of leptin. Physiol. Behav. 74, 703–708. 10.1016/S0031-9384(01)00613-8, PMID: 11790432

[ref22] Engelman J. A. Luo J. Cantley L. C. (2006). The evolution of phosphatidylinositol 3-kinases as regulators of growth and metabolism. Nat. Rev. Genet. 7, 606–619. 10.1038/nrg1879, PMID: 16847462

[ref23] Glastras S. J. Chen H. Pollock C. A. Saad S. (2018). Maternal obesity increases the risk of metabolic disease and impacts renal health in offspring. Biosci. Rep. 38:BSR20180050. 10.1042/BSR20180050, PMID: 29483369 PMC5874265

[ref24] Head G. A. Williams N. S. (1992). Hemodynamic effects of central angiotensin I, II and III in conscious rabbits. Am. J. Phys. Regul. Integr. Comp. Phys. 263, R845–R851. 10.1152/ajpregu.1992.263.4.R845 1415797

[ref25] Hirsch E. Costa C. Ciraolo E. (2007). Phosphoinositide 3-kinases as a common platform for multi-hormone signaling. J. Endocrinol. 194, 243–256. 10.1677/JOE-07-0097 17641274

[ref26] Huang E. J. Reichardt L. F. (2001). Neurotrophins: roles in neuronal development and function. Annu. Rev. Neurosci. 24, 677–736. 10.1146/annurev.neuro.24.1.677, PMID: 11520916 PMC2758233

[ref27] Kislal S. Shook L. L. Edlow A. G. (2020). Perinatal exposure to maternal obesity: lasting cardiometabolic impact on offspring. Prenat. Diagn. 40, 1109–1125. 10.1002/pd.5784 32643194 PMC7719098

[ref28] Klockener T. Hess S. Belgardt B. F. Paeger L. Verhagen L. A. Husch A.. (2011). High-fat feeding promotes obesity via insulin receptor/PI3K-dependent inhibition of SF-1 VMH neurons. Nat. Neurosci. 14, 911–918. 10.1038/nn.2847, PMID: 21642975 PMC3371271

[ref29] Lee K. K. Raja E. A. Lee A. J. Bhattacharya S. Norman J. E. Reynolds R. M. (2015). Maternal obesity during pregnancy associates with premature mortality and major cardiovascular events in later life. Hypertension 66, 938–944. 10.1161/HYPERTENSIONAHA.115.05920, PMID: 26370890

[ref30] Lim K. Barzel B. Burke S. Armitage J. Head G. (2016). The origin of aberrant blood pressure and sympathetic regulation in diet induced obesity. Hypertension 68, 491–500. 10.1161/HYPERTENSIONAHA.116.07461, PMID: 27296999

[ref31] Lim K. Burke S. L. Head G. A. (2013). Obesity related hypertension and the role of insulin and leptin in high fat fed rabbits. Hypertension 61, 628–634. 10.1161/HYPERTENSIONAHA.111.00705, PMID: 23339171

[ref32] Lurbe E. Aguilar F. Alvarez J. Redon P. Torro M. I. Redon J. (2018). Determinants of cardiometabolic risk factors in the first decade of life: a longitudinal study starting at birth. Hypertension 71, 437–443. 10.1161/HYPERTENSIONAHA.117.10529, PMID: 29358459

[ref33] Myers M. G. Cowley M. A. Münzberg H. (2008). Mechanisms of leptin action and leptin resistance. Annu. Rev. Physiol. 70, 537–556. 10.1146/annurev.physiol.70.113006.100707, PMID: 17937601

[ref34] NCD-RisC (2017). Worldwide trends in body-mass index, underweight, overweight, and obesity from 1975 to 2016: a pooled analysis of 2416 population-based measurement studies in 128.9 million children, adolescents, and adults. Lancet 390, 2627–2642. 10.1016/S0140-6736(17)32129-3, PMID: 29029897 PMC5735219

[ref35] Prior L. Davern P. Burke S. Lim K. Armitage J. Head G. (2014). Exposure to a high fat diet during development alters leptin and ghrelin sensitivity and elevates renal sympathetic nerve activity and arterial pressure in rabbits. Hypertension 63, 338–345. 10.1161/HYPERTENSIONAHA.113.02498, PMID: 24191287

[ref36] Prior L. Eikelis N. Armitage J. Davern P. Burke S. Montani J.-P.. (2010). Exposure to a high-fat diet alters leptin sensitivity and elevates renal sympathetic nerve activity and arterial pressure in rabbits. Hypertension 55, 862–868. 10.1161/HYPERTENSIONAHA.109.141119, PMID: 20194306

[ref37] Rios M. Fan G. Fekete C. Kelly J. Bates B. Kuehn R.. (2001). Conditional deletion of brain-derived neurotrophic factor in the postnatal brain leads to obesity and hyperactivity. Mol. Endocrinol. 15, 1748–1757. 10.1210/mend.15.10.0706, PMID: 11579207

[ref38] Shi Z. Li B. Brooks V. L. (2015). Role of the paraventricular nucleus of the hypothalamus in the Sympathoexcitatory effects of leptin. Hypertension 66, 1034–1041. 10.1161/HYPERTENSIONAHA.115.06017, PMID: 26370892 PMC4798233

[ref39] Simonds S. E. Pryor J. T. Ravussin E. Greenway F. L. Dileone R. Allen A. M.. (2014). Leptin mediates the increase in blood pressure associated with obesity. Cell 159, 1404–1416. 10.1016/j.cell.2014.10.058, PMID: 25480301 PMC4259491

[ref40] Taylor P. D. Matthews P. A. Khan I. Y. Rees D. Itani N. Poston L. (2018). Generation of maternal obesity models in studies of developmental programming in rodents. Methods Mol. Biol. 1735, 167–199. 10.1007/978-1-4939-7614-0_9 29380312

[ref41] Taylor P. D. Samuelsson A. M. Poston L. (2014). Maternal obesity and the developmental programming of hypertension: a role for leptin. Acta Physiol. 210, 508–523. 10.1111/apha.12223 24433239

[ref42] Toda C. Shiuchi T. Kageyama H. Okamoto S. Coutinho E. A. Sato T.. (2013). Extracellular signal-regulated kinase in the ventromedial hypothalamus mediates leptin-induced glucose uptake in red-type skeletal muscle. Diabetes 62, 2295–2307. 10.2337/db12-1629 23530005 PMC3712028

[ref43] Unger T. J. Calderon G. A. Bradley L. C. Sena-Esteves M. Rios M. (2007). Selective deletion of BDNF in the ventromedial and dorsomedial hypothalamus of adult mice results in hyperphagic behavior and obesity. J. Neurosci. 27, 14265–14274. 10.1523/JNEUROSCI.3308-07.2007, PMID: 18160634 PMC6673437

[ref44] Xu B. Goulding E. H. Zang K. Cepoi D. Cone R. D. Jones K. R.. (2003). Brain-derived neurotrophic factor regulates energy balance downstream of melanocortin-4 receptor. Nat. Neurosci. 6, 736–742. 10.1038/nn1073, PMID: 12796784 PMC2710100

[ref45] Yan X. Zhu M. J. Xu W. Tong J. F. Ford S. P. Nathanielsz P. W.. (2010). Up-regulation of toll-like receptor 4/nuclear factor-kappaB signaling is associated with enhanced adipogenesis and insulin resistance in fetal skeletal muscle of obese sheep at late gestation. Endocrinology 151, 380–387. 10.1210/en.2009-0849, PMID: 19887565 PMC2803146

[ref46] Zambrano E. Ibanez C. Martinez-Samayoa P. M. Lomas-Soria C. Durand-Carbajal M. Rodriguez-Gonzalez G. L. (2016). Maternal obesity: lifelong metabolic outcomes for offspring from poor developmental trajectories during the perinatal period. Arch. Med. Res. 47, 1–12. 10.1016/j.arcmed.2016.01.004, PMID: 26827819

